# Effects of an 8-Week Resistance Exercise Program on Body Composition and Lipid Profile in Women with Obesity

**DOI:** 10.3389/fphys.2026.1891546

**Published:** 2026-07-01

**Authors:** Tuna Turgut, Zülbiye Kaçay, Gamze Murathan, Laurentiu-Gabriel Talaghir, Bogdan Sorin Olaru, Daniel Mădălin Coja

**Affiliations:** 1 Faculty of Sport Sciences, Bartın University, Bartın, Türkiye; 2 Faculty of Sports Sciences, Çanakkale Onsekiz Mart University, Çanakkale, Türkiye; 3 Faculty of Sports Sciences, Adıyaman University, Adıyaman, Türkiye; 4 Faculty of Physical Education and Sport, Dunarea de Jos University of Galati, Galati, Romania

**Keywords:** body composition, lipid profile, metabolic health, obesity, resistance exercise

## Abstract

**Purpose:**

Obesity is a major global health concern associated with increased risk of metabolic and cardiovascular diseases. Resistance exercise has emerged as an effective non-pharmacological strategy for improving body composition and metabolic health; however, its effects may vary depending on individual and program-related factors. This study aimed to investigate the effects of an 8-week resistance exercise program on body composition and lipid profile in adult women with obesity.

**Methods:**

A total of 30 adult women (mean age: 28.97 ± 6.96 years) participated in an 8-week supervised resistance exercise program performed three times per week. The intervention was conducted at low-to-moderate intensity and progressively advanced through increases in exercise complexity, training volume, and external resistance. Body composition parameters were assessed using bioelectrical impedance analysis (BIA), and fasting blood samples were collected to determine triglycerides, total cholesterol, low-density lipoprotein (LDL), and high-density lipoprotein (HDL) levels. Pre- and post-intervention differences were analyzed using paired sample t-tests, and effect sizes (Cohen’s d) were calculated.

**Results:**

Significant improvements were observed following the intervention. Body weight (d = 0.69, p < 0.001), body fat percentage (d = 0.91, p < 0.001), and BMI (d = 0.78, p < 0.001) decreased significantly. Additionally, triglycerides (d = 1.25, p < 0.001), total cholesterol (d = 1.45, p < 0.001), and LDL levels (d = 1.25, p < 0.001) were significantly reduced, while HDL levels increased significantly (d = −0.70, p < 0.001). These findings indicate substantial improvements in both anthropometric and metabolic parameters.

**Conclusion:**

Participation in an 8-week resistance exercise program was associated with improvements in body composition and lipid profile parameters in women with obesity. However, due to the single-group pre-post design and the absence of a control group, causal relationships cannot be definitively established. Resistance exercise may represent a promising strategy for supporting cardiometabolic health in this population.

## Introduction

The World Health Organization (WHO) has recognized physical inactivity as a global public health problem and listed it among the global risk factors for death, along with hypertension and obesity (Akçay et al., 2023). Obesity is a complex and multifactorial condition characterized by excessive fat accumulation resulting from an imbalance between energy intake and expenditure, and its global prevalence continues to rise. This condition substantially increases the risk of chronic diseases, including sarcopenia, type 2 diabetes mellitus (T2DM), and cardiovascular disorders, thereby highlighting the urgent need for effective intervention strategies. Among non-pharmacological approaches, exercise has emerged as a key therapeutic tool, targeting both central and peripheral mechanisms involved in energy regulation, metabolic control, and overall physiological function. At the central nervous system level, exercise modulates reward pathways and appetite-regulating hormones, thereby influencing energy intake, mood, and cognitive processes (Zhang et al., 2024).

Although a large body of evidence demonstrates that exercise interventions can significantly reduce fat mass, increase energy expenditure, and improve metabolic health, public health policies still tend to prioritize dietary and pharmacological approaches over physical activity. For instance, major public health authorities such as the Centers for Disease Control and Prevention (CDC) continue to emphasize dietary strategies in obesity management frameworks (Centers for Disease Control and Prevention [CDC], 2024). This imbalance in policy emphasis may limit the full potential of exercise-based interventions in both the prevention and treatment of obesity.

Physical activity plays a central role in weight management (Jakicic et al., 2018) and is widely recognized as a cornerstone in reducing the prevalence of overweight and obesity (Reiner et al., 2013). In addition to its metabolic benefits, regular physical activity is associated with reduced risks of cardiovascular disease and cancer-related mortality (Saint-Maurice et al., 2019). Beyond physical health, exercise also exerts positive effects on psychological well-being. Meta-analytic evidence indicates that regular exercise is associated with lower levels of depression (Herring et al., 2012) and anxiety (Wipfli et al., 2008), improved quality of life (Chou et al., 2012), and enhanced body image perception (Campbell and Hausenblas, 2009), particularly in individuals with overweight or obesity (Carraça et al., 2021).

Physical inactivity, on the other hand, has been identified as a major contributor to the global burden of disease (Ammar et al., 2023) and is closely associated with insulin resistance and reduced oxidative capacity in skeletal muscle (Alibegovic, 2010). In contrast, regular physical activity has been shown to play a crucial role in the prevention and management of T2DM by improving glycemic control and reducing associated comorbidities (Arciero et al., 2013; Vanninen et al., 1992; Zierath et al., 1996; Pérez-Martin et al., 2001). However, the optimal exercise prescription—particularly in terms of duration, frequency, and intensity—remains a topic of ongoing debate, especially for individuals at risk of or diagnosed with T2DM. Moreover, it has been reported that improvements in insulin sensitivity may diminish rapidly following short periods of inactivity (Mikines et al., 1988; Vukovich et al., 1996).

Despite these well-established benefits, the appropriate exercise dose required to maximize metabolic adaptations remains unclear. Earlier guidelines have recommended moderate-intensity exercise performed 3–5 days per week for 45–60 minutes (Kulkarni et al., 1998; Basdevant et al., 1998). More recent evidence, however, suggests that higher-intensity exercise may produce greater cardiometabolic benefits and enhance insulin sensitivity (Malin et al., 2016; Jelleyman et al., 2015; Mezghani et al., 2022).

**Figure 1:**
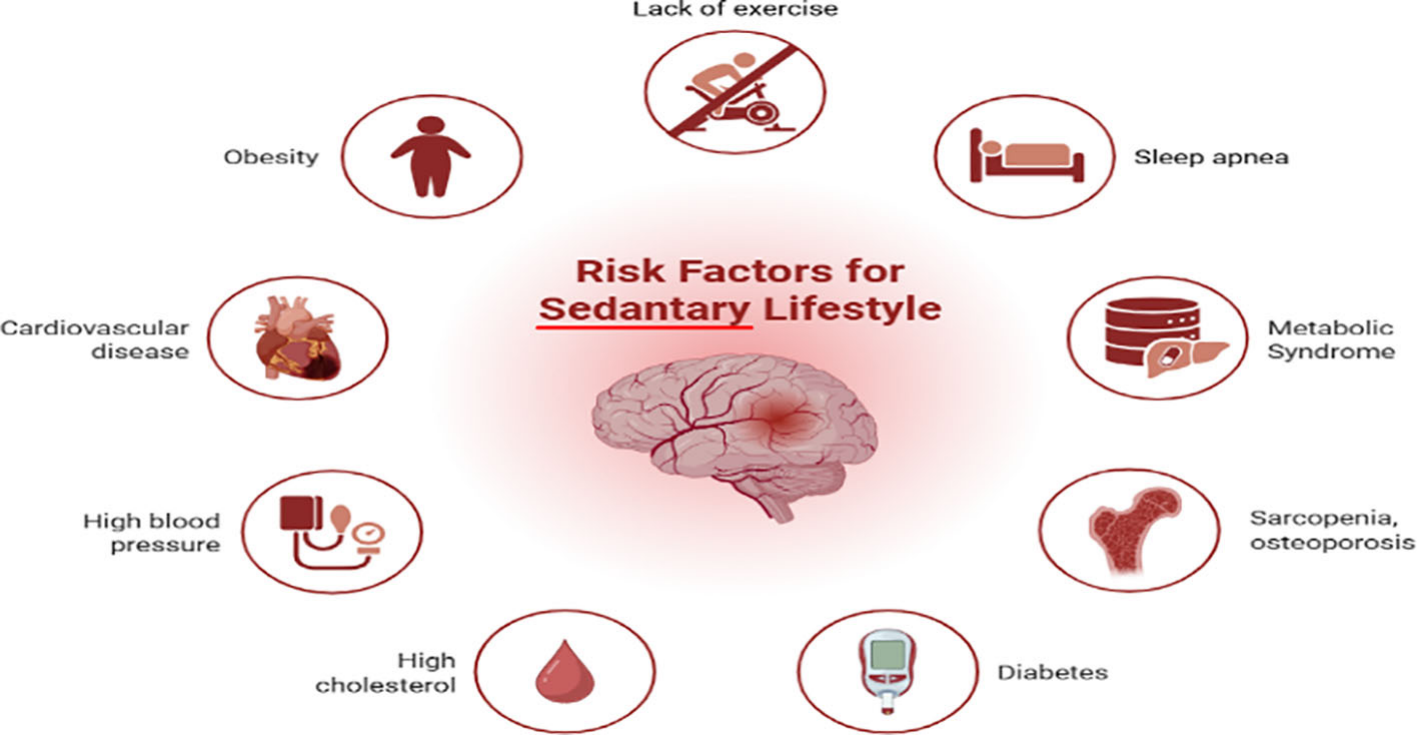
Illustrates the major health risks associated with physical inactivity and sedentary behavior.

**Figure 2:**
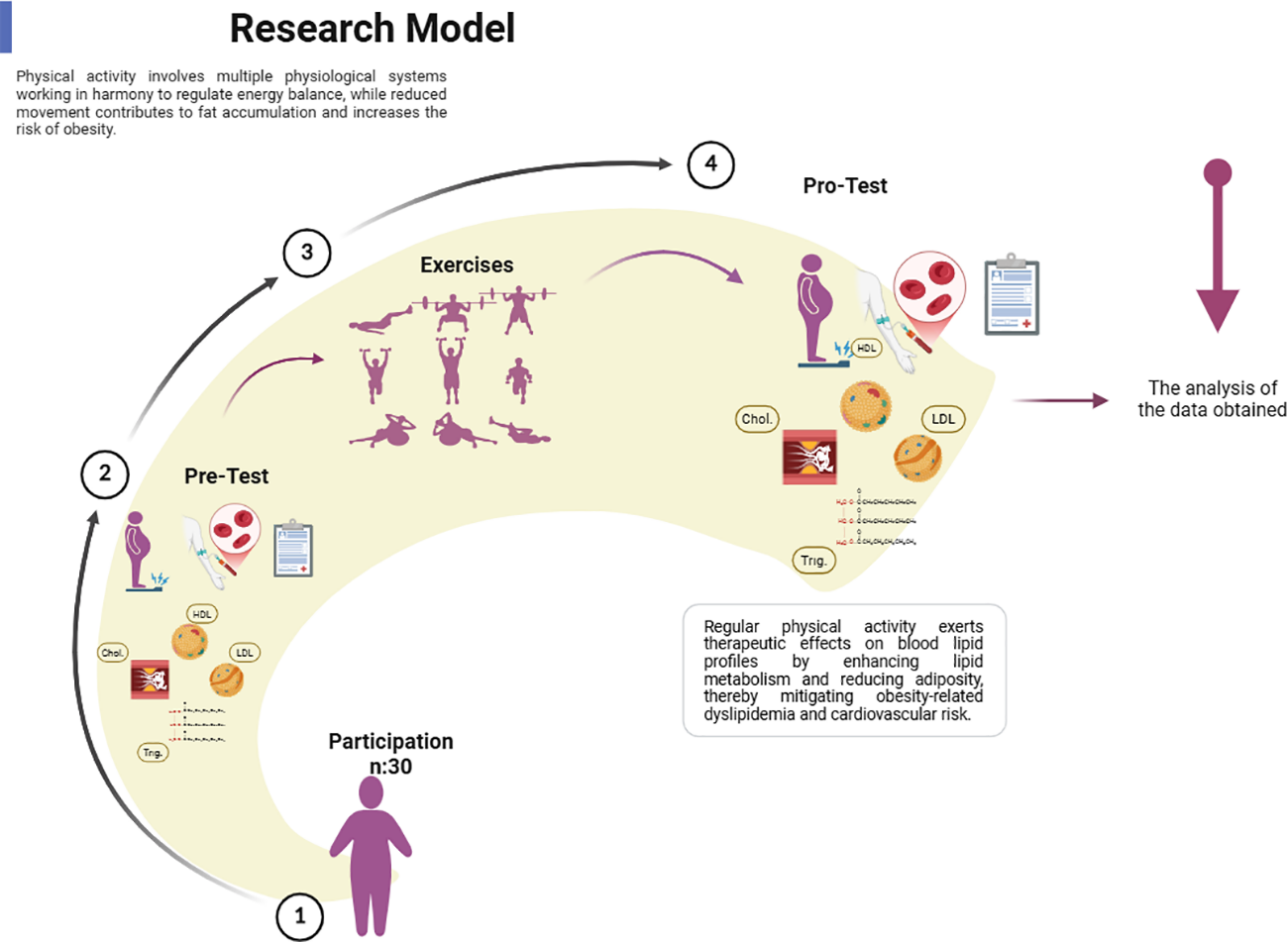
The study flow and assessment procedures.

Nevertheless, while the general benefits of exercise are well documented, there remains a need for further investigation into the specific effects of structured resistance exercise programs on both body composition and lipid metabolism, particularly in women with obesity. Therefore, the present study aimed to evaluate the effects of an 8-week resistance exercise intervention on body composition and lipid profile in adult women with obesity (Liu et al., 2024). The major health risks associated with physical inactivity and sedentary behavior, as well as the study flow and assessment procedures, are summarized in **Figures 1**, **2**.

## Methods

### Study design

This study employed a single-group pre-post intervention design (Büyüköztürk, 2016). Participants were assessed before and after an 8-week resistance exercise program to evaluate changes in body composition and lipid profile parameters. Because no control group was included, the study was designed to assess within-group changes over time rather than establish causal relationships between the intervention and the observed outcomes.

### Participants

*A priori* power analysis was conducted using G*Power (version 3.1) for paired-samples t-tests. Assuming a medium effect size (d = 0.70), α = 0.05, and statistical power of 0.80, a minimum sample size of 18 participants was required. Based on this calculation, a total of 30 volunteer adult women were recruited from a private fitness center.

Participants were instructed not to follow any specific dietary program and to refrain from engaging in additional structured exercise outside of the prescribed intervention. Prior to participation, all individuals were informed about the study procedures and provided informed consent.

The resistance exercise program was conducted three times per week (Monday, Wednesday, and Friday) at a fixed time (14:00–15:00) over an 8-week period.

### Inclusion and exclusion criteria

Participants were selected based on predefined inclusion and exclusion criteria. Inclusion criteria were as follows:

- women aged between 18 and 60 years;
- BMI between 25.0 and 39.9 kg/m²;
- absence of any medical condition preventing participation in exercise;
- no regular resistance exercise participation within the previous three months;
- voluntary participation in the study.

Exclusion criteria included:

- being younger than 18 or older than 60 years;
- BMI outside the range of 25.0–39.9 kg/m²;
- presence of cardiovascular, metabolic, orthopedic, or other health conditions limiting exercise participation;
- regular resistance training participation within the previous three months;
- unwillingness to participate voluntarily.

### Exercise intervention

Participants completed a structured resistance training program lasting 60 minutes per session, three times per week for 8 weeks. The program was progressively designed to improve muscular strength and body composition, with exercises targeting major muscle groups. All sessions were supervised to ensure proper technique and adherence to the protocol.

Training adherence and attendance were monitored throughout the intervention by recording participation at each supervised session. All participants completed the full intervention protocol, attending all 24 scheduled training sessions; therefore, the attendance and adherence rate was 100%. Exercise intensity was not prescribed based on one-repetition maximum testing because the participants were novice exercisers and the program prioritized safety, familiarization, and correct exercise technique. Instead, the intervention was performed at a low-to-moderate intensity, with progression achieved through increases in exercise complexity, training volume, and external resistance during weeks 5–8. Progression was implemented when participants were able to complete the prescribed sets and repetitions with proper technique, without pain, and without excessive fatigue.

### Body composition assessment

Body composition was assessed using bioelectrical impedance analysis (BIA) with a multi-frequency analyzer (Tanita BC-418, Tanita Corp., Tokyo, Japan). Measurements were performed under standardized conditions before and after the intervention period. Participants were instructed to attend the assessments in a fasted state and to avoid strenuous activity prior to measurement to ensure accuracy.

Parameters obtained included body weight, body fat percentage, and body mass index (BMI).

### Biochemical analysis

Biochemical measurements were obtained before and after the intervention. Participants provided fasting blood samples in the morning at a designated healthcare facility under standardized laboratory conditions. Blood analyses were performed by trained professionals using an automated analyzer (Beckman Coulter).

The following lipid profile parameters were assessed: triglycerides, total cholesterol, low-density lipoprotein (LDL), and high-density lipoprotein (HDL).

The resistance exercise program in **Table 1** was developed based on the recommendations of the American College of Sports Medicine (ACSM), international physical activity guidelines, and previously validated training protocols (Pelliccia et al., 2021). The intervention was structured progressively over the 8-week period.

**Table 1:** Structured resistance exercise program applied during the 8-week intervention.

Phase	Exercise	Sets × repetitions / duration
Phase (Weeks 1–4)	Warm-up (walking/cycling)	10 min
	Wall sit	2 x 15 s
	(Box squat)	2 x 12
	Modified push-ups	2 x 10
	Resistance band row	2 x 12
	Glute bridge	2 x 12
	Dumbbell shoulder press (light)	2 x 12
	Stretching and cool-down	5–7 min
Phase (Weeks 5–8)	Warm-up (light walking)	10 min
	Goblet squat	3 x 12
	Step-up	3 x 10
	Dumbbell bench press	3 x 10
	Bent-over row	3 x 10
	Single-leg glute bridge	2 x 10
	Plank progression	2 x 20 s
	Dumbbell lateral raise	2 x 12
	Stretching and cool-down	5–10 min

Bold values indicate the phase headings of the 8-week resistance exercise program.

During weeks 1–4, participants performed low-to-moderate intensity exercises focusing on familiarization and neuromuscular adaptation. Each session included a 10-minute warm-up (walking or cycling), followed by resistance exercises targeting major muscle groups: wall sit (2 × 15 s), box squat (2 × 12 repetitions), modified push-ups (2 × 10), resistance band rowing (2 × 12), glute bridge (2 × 12), and light dumbbell shoulder press (2 × 12). Sessions concluded with 5–7 minutes of stretching and cool-down.

During weeks 5–8, exercise intensity and volume were progressively increased. Sessions began with a 10-minute warm-up (light walking), followed by goblet squat (3 × 12), step-up (3 × 10), dumbbell bench press (3 × 10), bent-over row (3 × 10), single-leg glute bridge (2 × 10), plank progression (2 × 20 s), and dumbbell lateral raise (2 × 12). Each session ended with 5–10 minutes of stretching and cool-down exercises.

All sessions were supervised to ensure adherence, proper technique, and participant safety.

### Statistical analysis

Descriptive statistics were calculated and presented as mean ± standard deviation (SD). The normality of the data distribution was assessed using appropriate tests (e.g., Shapiro–Wilk test).

To evaluate the effects of the intervention, pre- and post-intervention differences were analyzed using paired sample t-tests. Effect sizes were calculated using Cohen’s d to determine the magnitude of the observed changes (Özmen and Karamustafaoǧlu, 2019).

The interpretation of effect sizes was based on established thresholds: <0.20 (trivial), 0.20–0.59 (small), 0.60–1.19 (moderate), 1.20–1.99 (large), and ≥2.00 (very large) (Hopkins et al., 2009).

The level of statistical significance was set at p < 0.05. All statistical analyses were performed using SPSS software (version 26.0; IBM Corp., Armonk, NY, USA). Graphical representations of pre- and post-intervention data were generated using JASP software (version 0.95).

## Results

Baseline anthropometric characteristics of the participants are summarized in **Table 2**. The mean age of the participants was 28.97 ± 6.96 years, with an average height of 163.9 ± 6.41 cm, body weight of 81.1 ± 9.11 kg, and BMI of 30.2 ± 2.47 kg/m². These values indicate that the study population consisted of adult women classified within the obesity range.

**Table 2:** Baseline characteristics of participants.

Variables	Mean ± SD
Age (year)	28.97 ± 6.96
Weight (kg)	81.1 ± 9.11
Height (cm)	163.9 ± 6.41
BMI (kg/m2)	30.2 ± 2.47

The distribution of participants across BMI categories before and after the intervention is presented in **Table 3**. Following the 8-week resistance exercise program, notable shifts in BMI classification were observed. The number of participants classified as normal weight increased to 8 (26.7%), whereas no participants were in this category at baseline. The overweight category increased from 9 (30.0%) to 19 (63.3%), reflecting a transition from higher BMI categories.

**Table 3:** Changes in BMI classification before and after the intervention.

Pre-Exercise			Post-Exercise		
BMI Classification	N	(%)	BMI Classification	N	(%)
Normal weight (BMI: <25)	0	0.0	Normal weight (BMI: <25)	8	26.7
Overweight (BMI: 25.0–29.9)	9	30.0	Overweight (BMI: 25.0–29.9)	19	63.3
Obesity class I (BMI: 30.0–34.9)	19	63.3	Obesity class I (BMI: 30.0–34.9)	3	10.0
Obesity class II (BMI: 35.0–39.9)	2	6.7	Obesity class II (BMI: 35.0–39.9)	0	0.0
Total	30	100	Total	30	100

The increase in the overweight category after the intervention reflects a transition of participants from higher BMI categories rather than a direct shift to normal weight. However, changes in BMI classification alone should be interpreted cautiously, as BMI does not directly reflect changes in body composition, metabolic health, or overall clinical status.

Conversely, the number of participants in the Obesity Class I category decreased substantially from 19 (63.3%) to 3 (10.0%), and those in Obesity Class II were no longer present post-intervention. The increase in the overweight category after the intervention reflects a transition of participants from higher BMI categories rather than a direct shift to normal weight. However, changes in BMI classification alone should be interpreted cautiously, as BMI does not directly reflect changes in body composition, metabolic health, or overall clinical status.

As shown in **Table 4**, significant improvements in body composition were observed following the intervention. Body weight decreased from 81.1 ± 9.11 kg to 74.2 ± 10.6 kg (p < 0.001, d = 0.69), indicating a moderate effect size. Similarly, body fat percentage decreased from 25.2 ± 2.18% to 22.3 ± 3.91% (p < 0.001, d = 0.91), corresponding to a moderate effect size.

**Table 4:** Changes in body weight, fat percentage, and BMI before and after the 8-week resistance exercise program.

Variables	Pre	Post	P	Cohen d	Magnitude
	Mean ± SD	Mean ± SD			
Weight (kg)	81.1 ± 9.11	74.2 ± 10.6	<0.001	0.69	Moderate
Body fat (%)	25.2 ± 2.18	22.3 ± 3.91	<0.001	0.91	Moderate
BMI (kg/m2)	30.2 ± 2.47	27.6 ± 4.0	<0.001	0.78	Moderate

BMI values also showed a significant reduction, decreasing from 30.2 ± 2.47 kg/m² to 27.6 ± 4.0 kg/m² (p < 0.001, d = 0.78), reflecting a moderate effect. Overall, these findings demonstrate that the resistance exercise program led to meaningful improvements in anthropometric parameters.

Changes in lipid profile parameters are presented in **Table 5**. Significant reductions were observed in triglycerides, total cholesterol, and LDL levels, while HDL levels increased significantly following the intervention.

**Table 5:** Changes in lipid profile before and after the eight-week resistance exercise program.

Variables	Pre	Post	P	Cohen *d*	Magnitude
	Mean ± SD	Mean ± SD			
Triglycerides*_(mg/dL)_*	165.9 ± 3.4	147.6 ± 12.7	<0.001	1.25	Large
Total Cholesterol*_(mg/dL)_*	201.4 ± 6.6	182.9 ± 7.5	<0.001	1.45	Large
HDL*_(mg/dL)_*	39.9 ± 3.7	43.2 ± 3.3	<0.001	-0.70	Moderate
LDL*_(mg/dL)_*	155.5 ± 17.2	126.6 ± 10.9	<0.001	1.25	Large

Triglyceride levels decreased from 165.9 ± 3.4 mg/dL to 147.6 ± 12.7 mg/dL (p < 0.001, d = 1.25), indicating a large effect size. Total cholesterol decreased from 201.4 ± 6.6 mg/dL to 182.9 ± 7.5 mg/dL (p < 0.001, d = 1.45), also representing a large effect. LDL levels decreased from 155.5 ± 17.2 mg/dL to 126.6 ± 10.9 mg/dL (p < 0.001, d = 1.25), indicating a very large effect size.

In contrast, HDL levels increased from 39.9 ± 3.7 mg/dL to 43.2 ± 3.3 mg/dL (p < 0.001, d = −0.70), corresponding to a moderate effect size. These findings suggest that the intervention resulted in substantial improvements in lipid metabolism and cardiometabolic risk markers.

**Figure 3:**
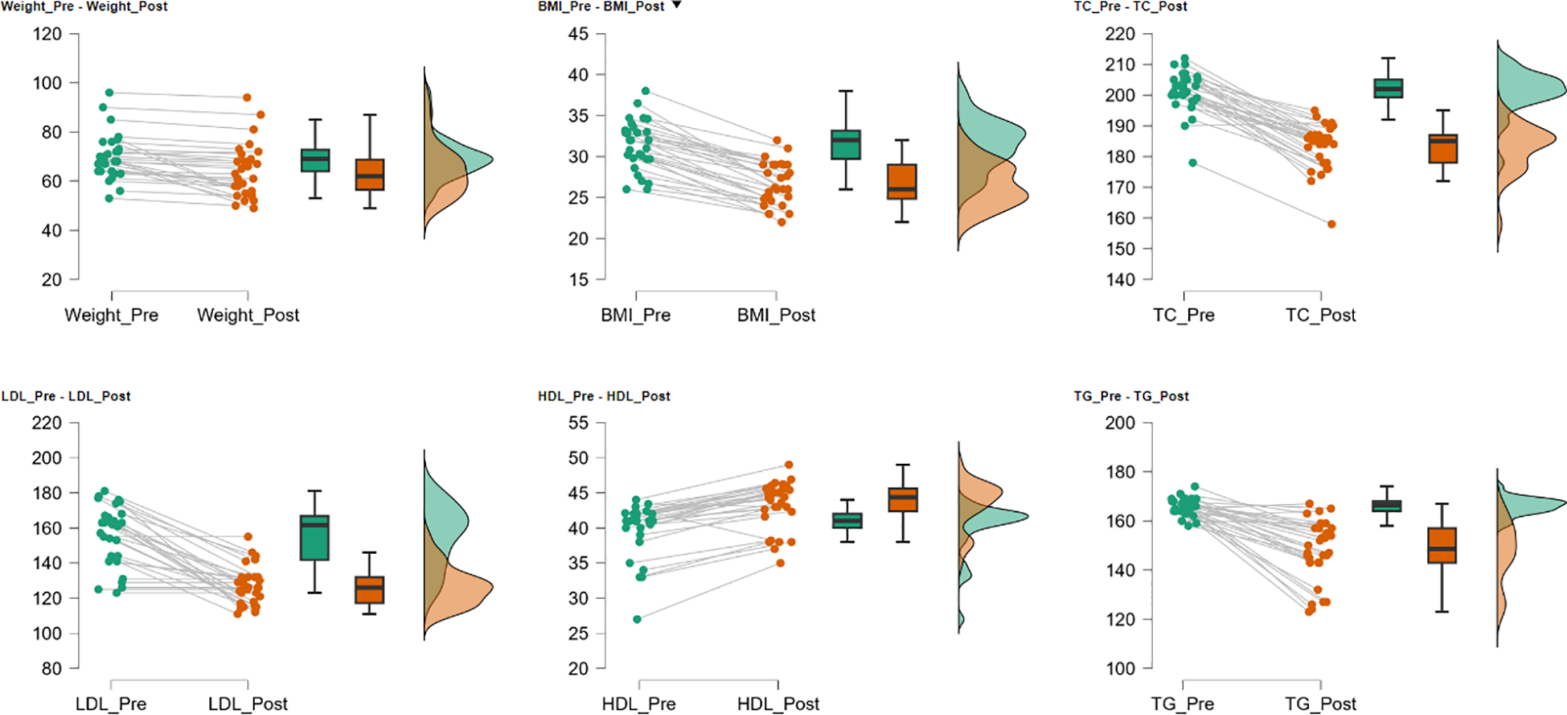
Changes in body composition and lipid profile before and after resistance exercise.

**Figure 3** illustrates both individual and group-level variations, demonstrating consistent improvements across most participants. Reductions in body weight, BMI, and body fat percentage are observed, alongside decreases in triglycerides, total cholesterol, and LDL levels, and an increase in HDL levels.

Individual paired data and distribution plots demonstrate consistent and clinically meaningful improvements across participants following the intervention.

**Figure 4:**
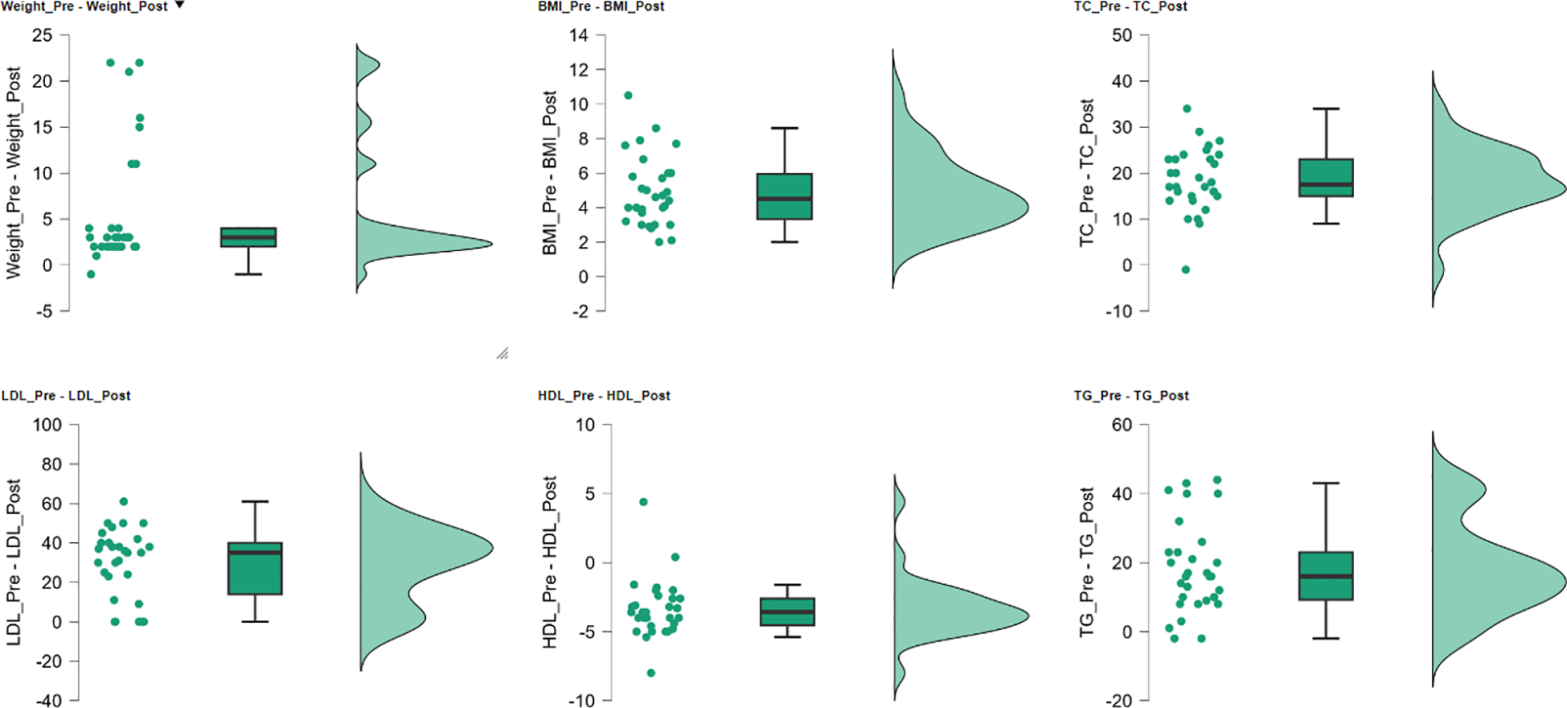
Effects of resistance exercise on body composition and lipid profile.

Graphical representations (**Figures 3**, **4**) illustrate both individual and group-level changes in body composition and lipid parameters. The paired data points demonstrate a consistent trend toward improvement across the majority of participants.

While reductions in body weight and BMI were moderate, changes in lipid parameters were more pronounced, particularly for LDL and total cholesterol. The observed patterns suggest that resistance exercise may contribute not only to reductions in adiposity but also to favorable alterations in metabolic health indicators.

## Discussion

The present study demonstrated that an 8-week resistance exercise program led to significant improvements in body composition and metabolic health indicators in adult women with obesity. The baseline characteristics of the participants, including age, height, and BMI, indicate a relatively homogeneous adult female sample, which strengthens the internal consistency of the findings. Moreover, these baseline characteristics highlight the relevance of physical activity interventions in similar populations, as emphasized by global health guidelines (World Health Organization, 2020).

One of the most notable findings of this study was the shift in BMI classification following the intervention. The increase in the number of participants within the normal weight category, along with the substantial reduction in obesity class I and II categories, suggests that resistance exercise contributes to a meaningful redistribution toward lower-risk BMI groups. Importantly, this shift reflects a gradual transition rather than an immediate normalization of body weight, indicating a progressive improvement in health status. These findings are consistent with previous epidemiological evidence highlighting the role of physical activity in promoting healthier BMI distributions and improving metabolic health outcomes (Jakicic et al., 2018; Swift et al., 2014).

The observed reductions in body weight, body fat percentage, and BMI further support the effectiveness of resistance training in improving body composition. The moderate effect sizes reported in this study indicate clinically meaningful changes. These findings are in line with previous research demonstrating that resistance-based exercise interventions can significantly reduce fat mass and improve overall body composition. For instance, a recent randomized controlled trial reported that a 12-week resistance training program improved body composition and reduced fat mass in sedentary overweight individuals (Ateş et al., 2024). Similarly, a systematic review focusing on older women with sarcopenic obesity concluded that resistance exercise is effective in reducing body fat percentage and improving body composition (Debes et al., 2024). These findings reinforce the robustness of the present results.

Although the reductions in body weight and BMI observed in the present study were statistically significant, the magnitude of these changes warrants careful interpretation. The average reduction of 6.9 kg over an 8-week period appears greater than that reported in many resistance-training interventions. Previous systematic reviews and meta-analyses have generally demonstrated modest reductions in body weight following resistance training alone, with more pronounced improvements typically observed in body composition variables rather than total body mass (Lopez et al., 2022; Wewege et al., 2022). Therefore, factors not directly monitored in the present study, including spontaneous dietary modifications, daily physical activity, and other lifestyle-related behaviors, may have contributed to the magnitude of the observed changes.

In addition to anthropometric improvements, significant changes in lipid profile parameters were observed. The reductions in triglycerides, total cholesterol, and LDL levels, along with the increase in HDL levels, indicate a favorable shift in lipid metabolism. The moderate to large effect sizes further suggest that these changes are not only statistically significant but also clinically relevant. These results are consistent with previous meta-analyses and randomized controlled trials reporting that resistance training can lead to significant improvements in lipid profiles (Kelley and Kelley, 2009). Furthermore, reductions in triglycerides and total cholesterol have been reported to be particularly pronounced in populations with elevated cardiometabolic risk (He et al., 2023).

However, it is important to note that the effects of resistance exercise on lipid profiles are not always consistent across studies. Earlier research has reported limited or non-significant changes in lipid parameters, particularly in individuals with coronary heart disease risk (Hurley, 1989). These inconsistencies may be attributed to variations in exercise type, duration, intensity, and baseline metabolic status of participants. Additionally, some studies have reported relatively modest changes in HDL levels, particularly in specific populations (He et al., 2023). Therefore, while the present findings support the beneficial effects of resistance exercise on lipid metabolism, the magnitude of these effects may vary depending on individual and program-related factors.

Taken together, the findings of this study suggest that resistance exercise represents an effective strategy for improving both body composition and lipid metabolism in women with obesity. However, the variability observed in the literature indicates that combining resistance exercise with other interventions, such as aerobic training and nutritional strategies, may further enhance metabolic outcomes.

However, the absence of a control group should be considered when interpreting the findings. Because the study employed a single-group pre-post design, the observed improvements cannot be attributed exclusively to the resistance exercise intervention. Uncontrolled factors such as spontaneous dietary changes, variations in daily physical activity, medication use, or other lifestyle-related influences may also have contributed to the observed outcomes. Therefore, the findings should be interpreted as changes observed following participation in the exercise program rather than definitive evidence of causality. Future randomized controlled studies with larger sample sizes and long-term follow-up periods are needed to better understand the sustainability and determinants of these adaptations.

## Limitations

Despite the significant findings of the present study, several limitations should be acknowledged. First, the study was conducted without a control group, which limits the ability to attribute all observed changes exclusively to the resistance exercise intervention. Second, the sample size was relatively small and included only adult women, which may limit the generalizability of the findings to other populations, including men and different age groups.

In addition, although participants were instructed not to follow a specific dietary program or engage in additional exercise during the intervention period, dietary intake and daily physical activity outside the exercise sessions were not objectively monitored. Therefore, potential lifestyle-related influences on the observed outcomes cannot be completely excluded. This limitation is particularly relevant when interpreting the substantial reductions observed in body weight and BMI, as unmeasured lifestyle factors may have influenced these outcomes.

Furthermore, medication use was not systematically monitored throughout the intervention period. Consequently, the potential influence of dietary modifications, pharmacological treatments, or changes in habitual physical activity on body composition and lipid profile outcomes cannot be ruled out. This limitation is particularly important when interpreting the substantial reductions observed in body weight and BMI.

Finally, body composition measurements were obtained using bioelectrical impedance analysis (BIA), which, although practical and widely used, may be less precise than gold-standard methods such as dual-energy X-ray absorptiometry (DEXA). Future studies involving larger and more diverse populations, controlled dietary monitoring, and randomized controlled designs are warranted to further clarify the effects of resistance exercise on metabolic health in individuals with obesity.

## Conclusion

The findings of the present study suggest that participation in an 8-week resistance exercise program was associated with significant improvements in body composition and lipid profile parameters in adult women with obesity. The observed effect sizes, ranging from moderate to very large, suggest that the intervention has substantial clinical relevance in terms of enhancing metabolic flexibility and overall homeostasis.

These results are consistent with the recommendations of major health organizations, including the World Health Organization (WHO, 2020) and the American College of Sports Medicine (Currier et al., 2026), which emphasize the importance of regular physical activity and resistance exercise in reducing the risk of chronic diseases. Accordingly, resistance exercise may represent a practical non-pharmacological approach that could support improvements in body composition and lipid profile parameters in women with obesity. However, given the absence of a control group, these findings should be interpreted cautiously.

### Practical implications

Regular resistance exercise programs should be encouraged as part of routine health strategies for obesity management and cardiometabolic risk reduction.

Future research should investigate the effects of resistance exercise across different age groups, sexes, and baseline body composition levels to enable more personalized intervention approaches.

Combining resistance exercise with aerobic training and nutritional interventions may yield greater improvements in metabolic health outcomes.

## Data Availability

The raw data supporting the conclusions of this article will be made available by the authors, without undue reservation.
